# Light-assisted electrospinning monitoring for soft polymeric nanofibers

**DOI:** 10.1038/s41598-020-73252-4

**Published:** 2020-10-01

**Authors:** Dario Lunni, Goffredo Giordano, Francesca Pignatelli, Carlo Filippeschi, Stefano Linari, Edoardo Sinibaldi, Barbara Mazzolai

**Affiliations:** 1 Center for Micro-BioRobotics, Istituto Italiano Di Tecnologia, Viale Rinaldo Piaggio 34, 56025 Pontedera, PI Italy; 2 The BioRobotics Institute, Scuola Superiore Sant’Anna, Viale Rinaldo Piaggio 34, 56025 Pontedera, PI Italy; 3 Department of Excellence in Robotics and AI, Scuola Superiore Sant’Anna, Piazza Martiri della Libertà 33, 56127 Pisa, PI Italy; 4 Linari Engineering S.R.L., Via Umberto Forti 24/14, 56121 Pisa, PI Italy

**Keywords:** Engineering, Nanoscience and technology

## Abstract

A real-time tool to monitor the electrospinning process is fundamental to improve the reproducibility and quality of the resulting nanofibers. Hereby, a novel optical system integrated through coaxial needle is proposed as monitoring tool for electrospinning process. An optical fiber (OF) is inserted in the inner needle, while the external needle is used to feed the polymeric solution (PEO/water) drawn by the process. The light exiting the OF passes through the solution drop at the needle tip and gets coupled to the electrospun fiber (EF) while travelling towards the nanofibers collector. Numerical and analytical models were developed to assess the feasibility and robustness of the light coupling. Experimental tests demonstrated the influence of the process parameters on the EF waveguide properties, in terms of waveguide length (L), and on the nanofibers diameter distribution, in terms of mean $\widehat{D}$ and normalized standard deviation $\chi$. Data analysis reveals good correlation between L and $\widehat{D}, \chi$ (respectively maximum correlation coefficients of ${\rho }_{L,\widehat{D}}$ = 0.88 and ${\rho }_{L,\chi }$ = 0.84), demonstrating the potential for effectively using the proposed light-assisted technology as real-time visual feedback on the process. The developed system can provide an interesting option for monitoring industrial electrospinning systems using multi- or moving needles with impact in the scaling-up of innovative nanofibers for soft systems.

## Introduction

In the last years one-dimensional (1D) nanostructures have gained much interest in research and industry because of their potential application in filtration^1^ for air purification, tissue engineering^2^, drug delivery^3^, sensors^4^, electronics^5^ and as smart materials for soft robotics^6–8^. More recent applications comprehend also Surface-enhanced Raman Scattering (SERS) sensing^9^, energy storage^10^ and photocatalysis^11–14^. Among the methods used to manufacture 1D nanostructures, like lithography^15^ or melt-blowing^16^ that are used to fabricate nanofibers membrane, electrospinning is particularly interesting because of its easiness to create complex nanostructures and potentialities to be scaled-up for industrial production^17^.

The electrospinning process is based on the elongation of a jet of a solution subject to an intense electrical field (10^5^–10^6^ V/m)^11^. The solution flow is controlled through a syringe pump endowed with a metallic needle, which is electrified during the process. When the applied voltage is high enough, the solution drop at the tip of the needle is stretched in the form of a cone shape, known as Taylor cone^18^. Extension thinning and/or thickening, and strain hardening of the jet takes place thanks to counteracting forces: the electrostatic force due to the accumulated charges, the surface tension, the gravitational force, the air drag force, and the viscoelasticity of polymeric liquids due to molecular entanglement, which strongly influences the strain and the strain rate^19,20^. At a critical level of voltage a jet of solution will start from the needle toward the conductive grounded collector and, if the molecular entanglement is high enough, the stream will not break down in sprayed droplets. In this way, the solution will flow through a droplet-jet transition region and a single fiber will accelerate. Due to the presence of charge of the same sign and solvent evaporation, the solution jet undergoes strong elongation and whipping motion that can reduce the fiber diameter to few hundreds nanometers. During the movement toward the collector, the solvent evaporates and thin fibers can be assembled on the grounded collector. If the process is stable, fibers can be deposited continuously until the solution flows and once the process stops dry samples can be removed from the collector for subsequent usage. The process is characterized by high versatility since virtually every soluble or fusible polymer can be used as precursor for fiber production. The polymer solutions can be modified adding particles, to obtain chemically active fibers^4^, or adding different polymers, to tailor the physical properties of the fibers membrane^21^. Also the mechanical setup can be easily customized to obtain ordered nanostructured devices, for example aligned or patterned nanofiber membranes can be fabricated by using a moving or structured collector^22^. Concentric spinnerets can be used for coaxial electrospinning^23–26^, eccentric spinneret can be implemented for side-by-side electrospinning^27^ or even more complex nanostructures can be obtained assembling tri-layer concentric spinneret^28,29^ or dedicated spinneret design^30^. The use of patterned or multilayered nanofibers membrane gives the possibility to tailor functionalized substrates for the particular application of the membrane.

Electrospinning shows promising scaling-up capability in terms of production volume, but the process is still affected by several issues concerning limited throughput, accuracy and reproducibility that do not allow for a complete industrial upscaling of the process^17^. The process is in fact strongly sensitive to ambient-dependent parameters (temperature, humidity level, electric field perturbations) that affect the reliability and the product quality and do not allow the reaching of the standard required for industrial applications^17,31^. Process monitoring, e.g., based on multiple process parameters, can permit to also increase throughput by preserving process outcome quality. Recent monitoring systems measure solution pressure^32^, current^33^ or even more parameters at the same time^34^ and often exploit such measurements inside closed-loop feedback system. Recently the transition region from droplet to jet, i.e. Taylor cone, was identified as crucial in electrospinning monitoring to predict nanofibers diameter and quality^35,36^. Taylor cone can be monitored using camera with zooming lens to do not interfere with electric field and to reduce arching probability^36^. In some cases, fluid velocity was estimated^37^ to investigate the jet dynamics of the process and by means of electrospinning models some prediction regarding the effect of the process parameters on the produced nanofibers can be done^38^. However, these models lack of accuracy, while monitoring systems are expensive and cannot easily track moving needles or multi-needle systems. Moreover, monitoring strategies using high-frame-rate cameras need image processing systems whose real-time implementation for closed-loop control would increase the cost and complexity of the system.

Within this framework and with the aim to improve process monitoring, we present in this paper a novel light-assisted electrospinning monitoring system. The developed setup is composed by a coaxial needle containing an optical fiber fastened in the inner needle, while the outer needle is used to feed the electrospinning solution. The underlying idea is the following, provided that outgoing light is guided towards the jet, we assumed that changes in the process control parameters could be reflected into waveguide properties (thus easily allowing for real-time visual feedback) in such a way to correlate process control parameters with the resulting electrospun fibers, in terms of some observable properties.

## Results

### Light-assisted electrospinning system overview

**Figure 1:**
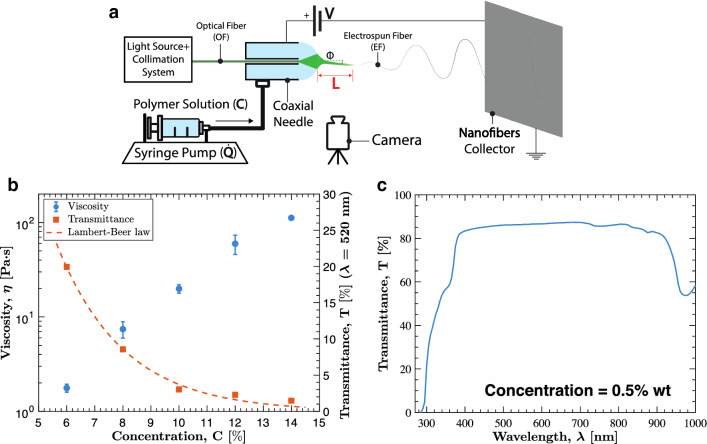
Light-assisted electrospinning system and solution characterization. (**a**) Schematized system comprising: optical fiber and source for light controlling, syringe pump and voltage generator for process control, coaxial needle, camera and nanofibers collector. (**b**) Solution viscosity and transmittance ($\lambda$ = 520 nm) characterization at different concentrations. (**c**) Transmittance of the solution at different wavelength.

System concept is schematized in Fig. 1a. The system is composed by a standard assembly for electrospinning (i.e., high voltage generator, syringe pump with metallic needle and nanofibers collector), a visible light source, and a lenses collimation system to collect light into a plastic optical fiber (OF). For simplicity, we considered a commercially available plastic OF made of PMMA. The syringe pump system leads the solution to the external needle of a coaxial system, while the inner needle is occupied by the OF. When the solution fills the external tank, a solution drop is formed at the tip of the needle and is lighten up by the assembled internal optical system. During the electrospinning process, a single fiber of material is drawn toward the collector, and the light exiting from the OF is expected to couple with the solution in the Taylor cone so as to enter the soft electrospun fiber (EF). As anticipated, we devised such a system based on the working assumption to be able to discriminate the effect of the chosen process control parameters on the resulting EF, based on a simple visual feedback. To the purpose, a CCD camera is introduced to record the EF, and in particular its initial segment, to obtain the length of light waveguide, labelled as L in Fig. 1a. The main process control parameters are the solution flow rate and the applied voltage, labelled as $\dot{Q}$ and $V$, respectively, in Fig. 1a.

### Polymer solution

The solution chosen to develop the setup is Polyethylene oxide (PEO) dissolved in water, because non-toxic and commonly used in electrospinning. The solution concentration, labelled as C in Fig. 1a, is known to affect conventional electrospinning because influencing viscosity and surface tension. A solution viscosity in the range 10^1^ ÷ 10^2^ Pa⋅s is usually desiderable^39^. Therefore, we firstly performed the viscosity characterization shown in Fig. 1b, thus obtaining 8% ÷ 12% wt as recommended concentration range for our PEO/water solution (see also “Materials and methods” section). The light passing through the solution partly gets absorbed depending on the chemical composition and concentration of the solution. Hence, we characterized light transmittance versus wavelength $\lambda$ as shown in Fig. 1c testing an almost transparent PEO/water solution at 0.5% wt with spectrophotometric analysis (see also “Materials and methods” section). Considering the chemical composition of the solution, the optimal transmittance wavelength window will not change significantly varying the solution concentration, but the transmittance value will decrease following the Lambert-Beer law (Fig. 1b). Considering the relatively flat trend for the maximum values of transmittance shown in the figure at hand, we adopted a LED source peaked in the visible spectrum at 520 nm for enlightening both the OF and EF.

### Model-based feasibility study

To assess the feasibility of the devised light-assisted electrospinning monitoring system we addressed light propagation to the EF by means of numerical and analytical approaches.

#### Numerical models

**Figure 2:**
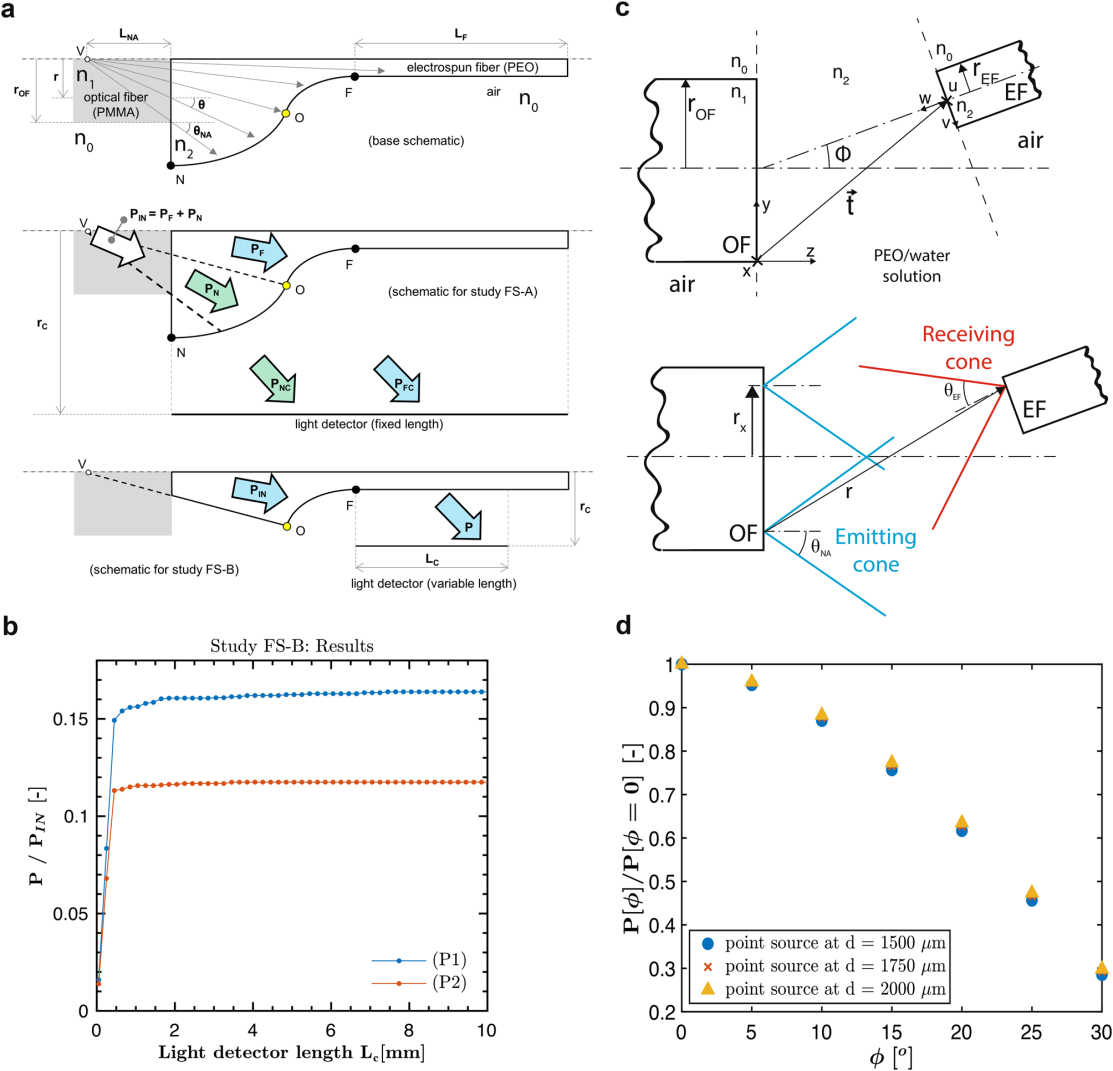
Model-based feasibility studies. (**a**) Schematics of the numerical feasibility studies. Up: base schematics showing the polymer solution (PEO) domain and the air domain. A conical light source at V is sketched, mimicking the light emitted from the PMMA-OF. Point O separates the drop-region (that one towards N) from the EF region (that one towards F). The curved profile in between N and F was determined based on experimental recordings of the drop-jet transition region; the rectangular region starting at F was added to account for the EF. Middle: schematics for study FS-A. The inlet power P_IN_ enters either through the drop region (P_N_) or through the EF region (P_F_). The study aimed at computing P_NC_/P_IN_ and P_FC_/P_IN_. Bottom: schematics from study FS-B. A fraction P of the inlet power P_IN_, traveling through the EF region, reaches the detector. The study aimed at computing P/P_IN_ as a function of the detector length L_C_. (**b**) Results of the numerical feasibility study FS-B: trend of the power fraction P/P_IN_ versus detector length L_C_, for both the experimentally derived droplet-jet transition profiles P1 and P2. (**c**) Schematics of the analytical model. Up: schematics showing the main domains of the model. The x–y–z frame is fixed at the lower corner of OF (that has radius r_OF_ and refractive index n_1_). The space between OF and EF (containing the PEO/water solution) is modeled as a domain with refractive index n_2_. The frame u–v–w is fixed on the EF (that has radius r_EF_ and refractive index n_2_) facet axis. The position and orientation of u–v–w are defined through the vector $\overrightarrow{t}$ and angle $\phi$ (respect to x–y–z). Both OF and EF are considered to be immersed in air (n_0_). Bottom: emitting (with $2{\theta }_{NA}$ apex angle) and receiving (with $2{\theta }_{EF}$ apex angle) cone on respectively OF and EF facets are shown. The represented ray r emitted from a generic emitting cone at distance r_x_ from the axis is considered accepted by the EF because reaching the facet inside the receiving cone. (**d**) Results of the analytical model: trend of the power fraction P($\phi$)/P($\phi$=0) varying the tilt angle $\phi$ considering a point source at different position behind the OF facet.

First, we focused on the possibility for light rays to enter the EF when it is aligned with the OF, based on the specific shape of the droplet-jet transition region. We considered two experimentally recorded transition regions, and we derived their profiles, hereafter labeled as P1 and P2, through image processing (see Supporting Information Fig. S1). By assuming axial symmetry, we modeled the droplet-jet transition region profile as in Fig. 2a (upper sketch). In particular, point N denotes the base radius of the drop, F denotes the beginning of the EF, and O separates the drop region from the EF region (further details on the domain geometry are reported in the “Materials and methods” section). For simplicity, light rays were assumed to originate at a point V at distance L_NA_ from the OF facet. Such a distance was derived from the OF radius (r_OF_ = 250 µm), the OF numerical aperture (NA), dependent on the refractive indices of air (n_0_), OF (n_1_), and on the refractive index of the EF (n_2_). The source results in a maximum emitting cone with apex angle equal to 2 ${\theta }_{NA}$ (further details on the optical parameters are reported in the “Materials and methods” section).

In the first feasibility study (FS-A, see the middle sketch in Fig. 2a), we introduced an in silico light detector parallel to the axis (r_c_ = 5 cm) for the entire computational domain to mimic a side visual inspector. Given an arbitrary input power P_IN_, we then computed the power fractions P_NC_/P_IN_ and P_FC_/P_IN_ reaching the detector by passing through the droplet (P_NC_) and the EF (P_FC_) region, respectively. For profile P1 we obtained P_NC_/P_IN_ = 87.5% and P_FC_/P_IN_ = 1.1%; for profile P2 we obtained P_NC_/P_IN_ = 89.7% and P_FC_/P_IN_ = 0.7%. While showing that the great majority of the power flux passes through the drop, this preliminary estimate suggested the possibility to discriminate EF illumination in between P1 and P2, based on the relative increase (around 60%) in the corresponding detected power. In order to further investigate this issue, we carried out a second feasibility study (FS-B, see the lower sketch in Fig. 2a), where we introduced an in silico detector (r_c_ = 0.5 mm) having a variable length L_C_. In such a second study we only considered an arbitrary input power P_IN_ through the EF region, and we computed the detected power fraction P/P_IN_(L_C_), i.e. as a function of the detector length L_C_. This way, we aimed at assessing the decay of light emission along the EF. The obtained results, reported in Fig. 2b, showed that profile P1 emitted sensibly more power than P2 (above 0.16/0.12 ≅ 133%), over a longer EF span. Indeed, while for P2 light emission practically ended in roughly 1 mm, for P1 it extinguished on the order of 10 mm. Based on the results obtained from both numerical models, we deemed it feasible to visually discriminate two different droplet-jet transition regions based on the corresponding diffused light.

#### Analytical models

Before prototyping the system, we also assessed the potential effect of a misalignment and tilting between OF and EF. To the purpose, we developed a simplified analytical model, based on the one introduced in Taylor et al.^40^ to estimate losses in fiber optic joints due to misalignments. More in detail, we generalized the model implementing the EF rotation and translation on a 2D plane. With the reference to Fig. 2c, given the angle ϕ between OF and EF axes, the introduced model was aimed at estimating the power loss as a function of ϕ, namely P(ϕ)/P(ϕ = 0), where P denotes the power entering EF. The bare emitting fiber (OF) with radius r_OF_ and refractive index n_1_, is surrounded by air, with refractive index n_0_. An interlayer spacing (representing the droplet-jet transition region) was defined as infinite domain with a fixed refractive index (n_2_), resulting an emitting cone with $2{\theta }_{NA}$ as apex angle (Fig. 2c). The bare electrospun receiving fiber (EF) with radius (r_EF_ ), refractive index (n_2_) and surrounded by air (resulting with a receiving cone with $2{\theta }_{EF}$ as apex angle), was tilted at different angles (ϕ) spanning from 0° to 30°. The obtained results, reported in Fig. 2d, showed that a considerable power fraction (30%) would be transmitted even for misalignment angles as large as 30° for point sources (placed at different positions behind the OF plane on the axis), which is a typically satisfied working condition^41^. An accurate description for a point source accounting also longitudinal misalignments is reported in Figs. S6.1, S6.2, S6.3. A droplet-jet transition region length around one order larger than the OF radius allows in the first instance to consider only point sources (far-field). At far-field, all the rays exiting within the OF maximum emitting cone encountering the EF will be accepted within the EF maximum acceptance cone. Indeed, only in extremal cases a fraction of the rays exiting the OF are considerably not accepted, for example when the droplet-jet transition width is below the OF radius (near-field) and a sensitive longitudinal misalignment lead off (as shown in Supporting Information Figs. S6, S7). The devised monitoring system, therefore, can be also robust with respect to disturbances/instabilities causing jet angular deviations (illustrated by picture in Supporting Information Fig. S2).

### Testing setup and mechanical assembly of the coaxial needle

**Figure 3:**
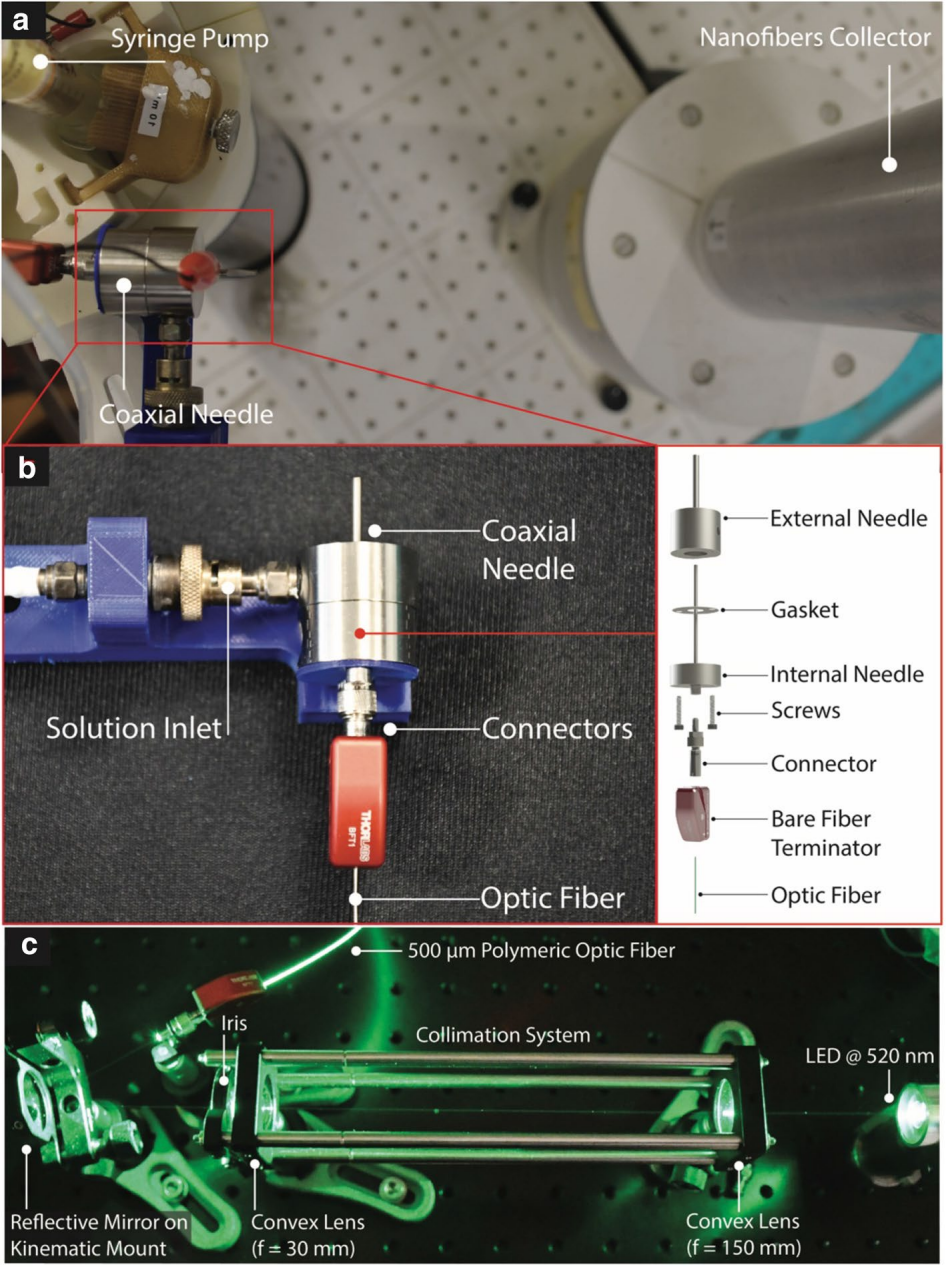
Experimental setup. (**a**) Experimental setup used to perform the experimental validation comprising syringe pump, coaxial needle and nanofibers collector. (**b**) Detailed representation of the realized coaxial needle and mechanical exploded view (inset). (**c**) Optical setup composed by light source, collimation system, reflective mirror and optical fiber (OF).

The testing setup was assembled as depicted in Fig. 3a. The coaxial needle is connected to the syringe pump for the solution feeding and is pointed towards the nanofibers collector placed at 20 cm distance. As depicted in Fig. 3b, the coaxial steel needle is composed by an external needle that is connected to the solution inlet and by an internal one that hosts the OF. The external needle is connected, through screws and sealed with a gasket, to the internal needle (Fig. 3b-inset). The OF is axially inserted until the tip of the fiber reaches the needle tip, and it is fixed with a bare fiber terminator located at the base of the system. The assembled coaxial needle was then connected to a voltage generator through a metallic clip visible in Fig. 3a. The optical setup used to enlighten optical fiber is shown in detail in Fig. 3c, the light coming from the LED@520 nm passes through a collimation system used to reduce the spot dimension nearly to 500 µm using two convex lenses. The iris allows the verification of the correct alignment of the setup. Finally, a reflective mirror mounted on a two degrees of freedom kinematic support allows the redirection of the beam toward the OF.

### Experimental assessment

**Figure 4:**
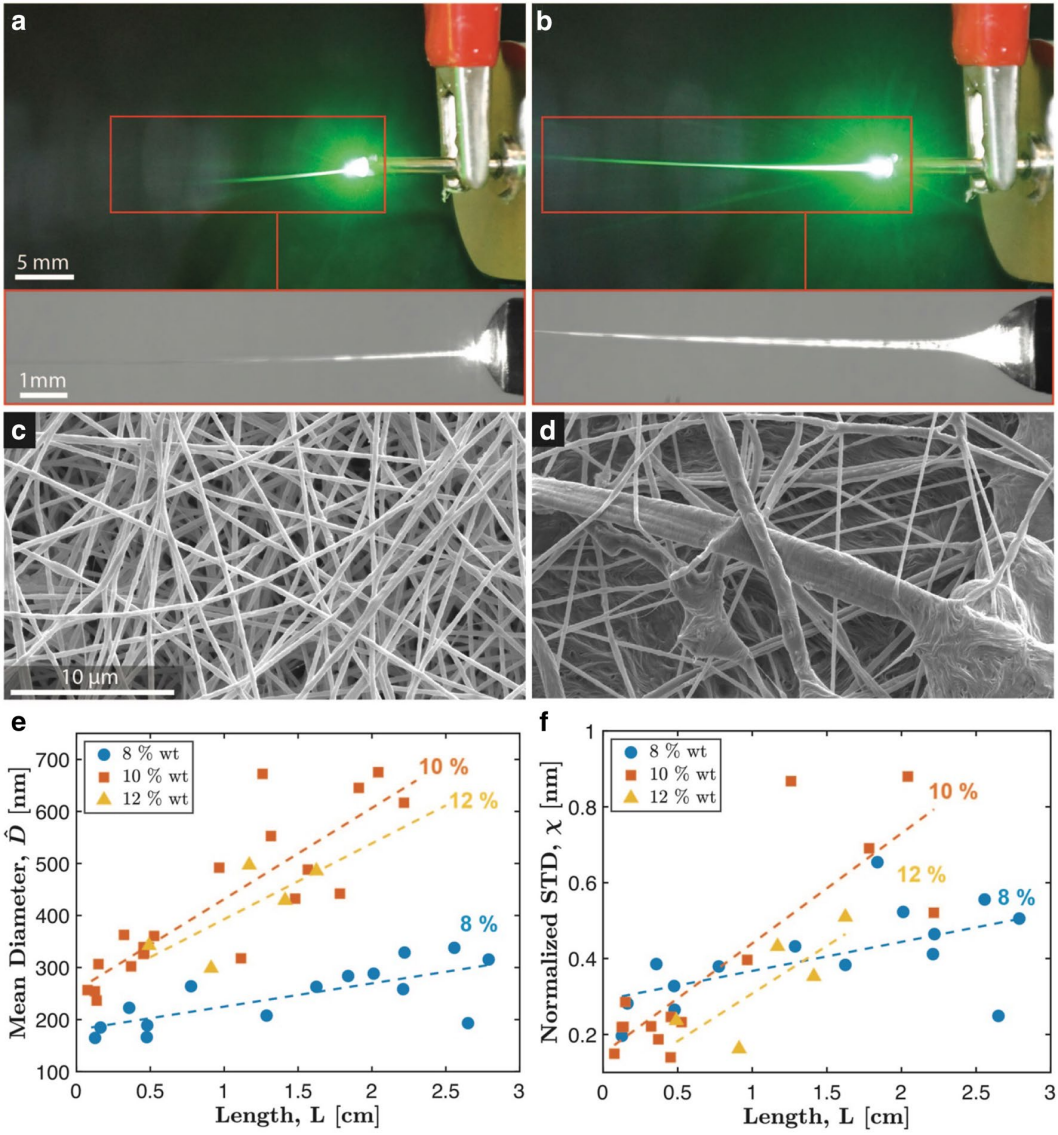
Experimental validation. (**a**,**b**) Two illuminated EFs associated with different control parameters. Different control parameters develop effects on the droplet-jet transition region affecting the light waveguide length during the process (insets). (**c**,**d**) Resulting EFs obtained through different process parameters observed through SEM. (**e**,**f**) Plot of the EFs distribution descriptors ($\widehat{D}, \chi$) as function of the observable variable ($\widehat{L}$). Linear regression model are shown underlying the linear monotonic trends.

Figure 4a,b show two examples of illuminated EFs, associated with different process control parameters. The figures at hand aim at illustrating the effect of the chosen control parameters on the droplet-jet transition region, and in particular on the light waveguide length (Fig. 4a,b-insets). The control parameters associated with Fig. 4a, in particular, are the same as those associated with Fig. S1 (i.e., profile P2), while those associated with Fig. 4b correspond to the ones associated with Fig. S1 (i.e., profile P1). Therefore, profile P1 turned out to be brighter than profile P2, and this is consistent with the numerical results in Fig. 2b, up to the many simplifications introduced for building the numerical model. Moreover, control parameters also reflected into different resulting EFs, as shown in Fig. 4c,d, respectively associated with Fig. 4a,b. An exhaustive illustration of the resulting waveguide length and EFs is reported in Figs. S3, S4, S5 (Supporting Information). Figure 4a,d suggest the possibility to assess process outcome by considering the distribution of the obtained EFs, in particular with respect to the fibers diameter through the normalized standard deviation (χ = ${\sigma }_{D}$/$\widehat{D}$). Tables 1, 2, 3 reports the waveguide length (L, mean $\widehat{L}$ ± std) by varying the control parameters, namely voltage (V), flow rate ($\dot{Q}$) and concentration (C), in the range of spinnability. More specifically, Tables 1, 2, 3 reports L for selected C values, by sweeping on V and $\dot{Q}$. We also quantified the EF diameter (D, mean $\widehat{D}$ ± std ${\sigma }_{D}$) for each electrospun sample, as obtained by applying image-processing to SEM images. The obtained results for EF diameter are included in Tables 4, 5, 6 and show a variation in the nanofibers diameter distribution varying the control parameters (Each mean value is reported with the standard deviation). By remapping the data presented in all the Tables, the plot in Fig. 4e,f are obtained, which show $\widehat{D}$ and χ of the diameter distribution versus the $\widehat{L}$ parameter.

**Table 1:** Mean L ($\widehat{L}$) for 8% wt concentration of PEO/water solution sweeping on V and $\dot{Q}$.

8% wt, Waveguided Length, L [cm]					
V [kV]	Solution flow, $\dot{Q}$ [ml/h]				
	1.0	2.0	3.0	4.0	5.0
20.0	0.12 ± 0.02	0.48 ± 0.08	0.16 ± 0.04	N/A	N/A
22.5	1.30 ± 0.15	2.21 ± 0.12	2.65 ± 0.13	N/A	N/A
25.0	0.48 ± 0.05	1.84 ± 0.08	2.01 ± 0.12	2.79 ± 0.04	3.14 ± 0.18
27.5	0.36 ± 0.17	0.78 ± 0.05	1.62 ± 0.08	2.22 ± 0.09	2.56 ± 0.10

**Table 2:** Mean L ($\widehat{L}$) for 10% wt concentration of PEO/water solution sweeping on V and $\dot{Q}$.

PEO/water solution 10% wt, Waveguided Length, L [cm]						
V [kV]	Solution flow, $\dot{Q}$ [ml/h]					
	1.0	2.0	3.0	4.0	5.0	6.0
22.5	0.08 ± 0.02	0.13 ± 0.04	0.14 ± 0.04	0.15 ± 0.03	N/A	N/A
25.0	N/A	0.37 ± 0.07	0.32 ± 0.04	0.53 ± 0.06	0.45 ± 0.07	N/A
27.5	0.45 ± 0.04	0.37 ± 0.25	1.26 ± 0.26	1.79 ± 0.14	2.04 ± 0.10	2.22 ± 0.14
30.0	0.4 ± 0.09	1.11 ± 0.06	1.48 ± 0.07	1.32 ± 0.07	1.57 ± 0.10	1.91 ± 0.21

**Table 3:** Mean L ($\widehat{L}$) for 12% wt concentration of PEO/water solution sweeping on V.

PEO/water solution 12% wt, Waveguided Length, L [cm]	
V [kV]	Solution flow, $\dot{Q}$ [ml/h]
	1.0
25	0.49 ± 0.08
27.5	0.91 ± 0.03
30	1.17 ± 0.23
32.5	1.62 ± 0.04
35	1.41 ± 0.12

**Table 4:** Mean $D$ ($\widehat{D}$) for 8% wt concentration of PEO/water solution sweeping on V and $\dot{Q}$.

PEO/water solution 8% wt, Fibers Diameter, D [nm]					
V [kV]	Solution flow, $\dot{Q}$ [ml/h]				
	1.0	2.0	3.0	4.0	5.0
20,0	164 ± 32	166 ± 54	185 ± 52	N/A	N/A
22,5	207 ± 89	258 ± 106	193 ± 48	173 ± 74	N/A
25,0	188 ± 50	283 ± 185	288 ± 151	315 ± 160	N/A
27,5	222 ± 85	264 ± 100	263 ± 101	329 ± 153	338 ± 188

**Table 5:** Mean $D$ ($\widehat{D}$) for 10% wt concentration of PEO/water solution sweeping on V and $\dot{Q}$.

PEO/water solution 10% wt, Fibers Diameter, D [nm]						
V [kV]	Solution flow, $\dot{Q}$ [ml/h]					
	1.0	2.0	3.0	4.0	5.0	6.0
22.5	257 ± 38	254 ± 56	237 ± 53	306 ± 88	N/A	N/A
25.0	N/A	302 ± 57	363 ± 81	361 ± 84	326 ± 46	N/A
27.5	339 ± 84	492 ± 195	672 ± 584	442 ± 306	675 ± 595	617 ± 322
30.0	328 ± 68	432 ± 122	553 ± 346	489 ± 273	513 ± 291	390 ± 152

**Table 6:** Mean $D$ ($\widehat{D}$) for 12% wt concentration of PEO/water solution sweeping on V.

PEO/water solution 12% wt, Fibers Diameter, D [nm]	
V [kV]	Solution flow, $\dot{Q}$ [ml/h]
	1.0
25.0	342 ± 81
27.5	299 ± 49
30.0	497 ± 215
32.5	486 ± 248
35.0	429 ± 152

Firstly, a correlation of the chosen control parameters ($C, V, \dot{Q}$) and the resulting observable variables ($\widehat{D}, {\sigma }_{D}, \widehat{L}$) was investigated. The linear correlation coefficients are reported in Supporting Information and show, as expected, a correlation between the control variables and the observable variables. Also multivariate regression models were investigated (considering second degree polynomial regression) and the best fit is reported in Supporting Information.

Secondly, correlation coefficients among the observable parameter $\widehat{L}$ and the description of the nanofibers EF distribution ($\widehat{D}$ and $\chi$) were investigated for each concentration. The linear correlation coefficients for each concentration ${{\rho }^{8\%}}_{\widehat{L},\widehat{D}}$= 0.73, ${{\rho }^{10\%}}_{\widehat{L},\widehat{D}}$= 0.88, ${{\rho }^{12\%}}_{\widehat{L},\widehat{D}}$= 0.73 show good positive correlation between $\widehat{L}$ and $\widehat{D}$ and a monotonic trend is observed among the considered variables. Also the correlations between $\widehat{L}$ and $\chi$ were investigated resulting in the correlation coefficients: ${{\rho }^{8\%}}_{\widehat{L},\chi }$= 0.58, ${{\rho }^{10\%}}_{\widehat{L},\chi }$= 0.84, ${{\rho }^{12\%}}_{\widehat{L},\chi }$= 0.79 which show good positive correlation between the observed variables and nanofibers descriptor. Linear regression models represented in Fig. 4e,f are also showing monotonic trends among the observed variables $\widehat{L}$ and the EF distribution descriptors ($\widehat{D}$ and $\chi$) suggesting the possibility to extract information from the process by observing $\widehat{L}$. Complete regression models with relative ${R}^{2}$ and p-Values for each considered parameter, demonstrating the significance of the regression, are reported in Supporting Information.

## Discussion

The correlation and linear trend exhibited by the obtained data confirm the hypothesis that the proposed approach represents an interesting option to implement optical and photonic technologies, to improve the monitoring of the electrospinning procedure. Based on the obtained results, the working assumption of a correlation between electrospinning control parameters and visible waveguide length was confirmed. In particular, an increase of the light waveguide length turned out to be positively correlated with the dispersion of the fibers diameter, and therefore with the potential presence of defects in the process outcome.

The proposed light-assisted system provides a flexible and cost-effective solution for monitoring electrospinning in real-time. Indeed, standard optical fibers and cameras can be used, without the need of optical zooming (e.g., to accurately track the droplet-jet transition region): visual-feedback based on the visible waveguide length offers a direct indicator useful for potential diagnosis by a human operator or an automatic system. Moreover, the add-ons associated with the proposed visual feedback permit to easily track moving needles or multi-needle systems, functional to flexible/reconfigurable manufacturing, while also avoiding any problems that zooming systems would face when integrated with movement and vibration control units.

We demonstrated the potential for seamless integration of optical technologies into electrospinning systems. To the purpose, we also leveraged simplified modeling techniques to preliminarily assess feasibility. Physical models could be further improved in order to provide an exhaustive characterization of the system. For instance, we adopted geometrical optics approaches for simplicity, however the changing physical properties in the droplet-jet transition region add to the complexity of light-matter interaction. The soft matter properties of the EF do not allow a remarkable effective L, because the longitudinal components of the electric field propagating into the optical fiber are not negligible, and outstanding radiation are scattered and exit at the EF-space interface^42^. Also a more accurate estimation of the optical losses would thus require a very detailed thermo-optical characterization, the implementation of Fresnel equation at the interface between different media and to consider the effect of absorbance along the fiber, aspects that would go beyond the scope of the present study. Nevertheless, our simplified model-based bootstrap turned out to be effective in steering the development of the physical system up to successful demonstration, and it could serve to apply the proposed approach to different combination of media allowing easily implementation into more complex assembly.

Regarding the considered process parameters, the hereby proposed study considers the control of solution flow, solution concentration and applied voltage, but fine tuning of the electrospinning process could be further developed based on advanced process characteristics such as Taylor cone’s height^35^ or length of the straight fluid jet^43,44^. Also deeper investigation about the regression model used should be investigated in order to improve the prediction capability of the proposed tool. In fact, the main focus of the study is related to the observation of a monotonic trend between the nanofibers characteristics (in terms of diameter) and the observable illuminated length. The trend has been characterized through a correlation analysis to quantitatively relate the considered variables, but a deeper analysis of this aspect (which goes beyond the scope of the present study) could advance the proposed monitoring strategy. In particular, an investigation on the dependence of the correlation degree with the concentration could improve the prediction accuracy. Anyhow, the observed monotonicity shows the possibility to use the illuminated length to extract information from the process. To integrate the proposed tool in an industrial design, an initial calibration phase of the system should be performed, based on the specifically adopted polymer solutions. Such a calibration phase would be necessary in order to predict the process outcome in terms of nanofibers diameters and their relationship with the observable illuminated length. Based on this calibration, the illuminated length could be used to obtain insights on the ongoing process, and differences in such parameter values with respect to the expected ones would be considered as anomalies of the industrial process status, requiring attention. The possibility to recapitulate process status through an inexpensive visual feedback opens up the possibility to develop flexible electrospinning monitoring systems, as functional, e.g., to large-scale fiber production, for example using multi-needle or moving needle systems also functional to increase process throughput. This can foster rising concepts and applications in soft robotics, e.g., for flexible sensors^45^ and actuators^7,8^. Moreover, accurate deposition of soft polymeric fibers could also foster further applications in organic electronics^46^, photonics^5^ and filtering application^47^. To wrap-up, the proposed light-assisted electrospinning monitoring systems could advance a variety of emerging technologies and applications, with potential impact on both industry and research.

## Conclusions

In summary, the paper presents an innovative integrated optical system proposed as monitoring tool for electrospinning process. A coaxial system endowed with an OF in the inner needle is assembled to couple the light exiting the OF with the EF. The process parameters such as $V, \dot{Q}$ and $C$ influence the process results in terms of diameter nanofibers distribution ($\widehat{D}, \chi$) but also the waveguide properties of the EF, in terms of waveguide length ($\widehat{L}$). Correlations between the observable parameter $\widehat{L}$ and ($\widehat{D}, \chi )$ are found, suggesting the possible usage of the proposed optical system as a real-time monitoring tool. Such solution would allow a real-time visual feedback on the process condition simplifying the monitoring task.

## Materials and methods

### Polymer solution preparation and characterization

Polyethylene oxide (PEO) was purchased from Sigma Aldrich and used without further purification. The molecular weight was 300,000 g/mol. PEO aqueous solutions with concentration of 6%, 8%, 10%, 12% and 14% in weight (PEO weight/solution weight) were used. Solutions were prepared mixing polymer with water and keeping them on an orbital shaker for 48 h before testing. Anton Paar MCR 302 modular compact rheometer was used for the rheological characterization of the PEO aqueous solutions using a double plate (25 mm diameter) setup. To ensure the repeatability of the measurement a fixed amount of volume 1 ml was used for each test. The considered viscosity was obtained once reached the plateau sweeping the rotational speed condition and considering a shear-thinning solution. UV−vis absorption spectra were measured with a PerkinElmer LAMBDA 45 spectrophotometer. For the measurements 3 ml PEO aqueous solutions, at different concentrations, were kept in disposable optical PS macro cuvettes, 10 mm light path, and the transmittance between 270 and 1000 nm was recorded.

### Coaxial needle

The parts assembling the coaxial needle were realized using traditional manufacturing techniques modifying commercial components (COAX-2DISP, Linari Engineering). PTFE gasket was laser cut from a 100 µm thick tape to ensure the absence of leakage of solution. The internal needle held the 500 µm diameter PMMA-OF in position during the testing, while the bare optical fiber terminator clip was fixing the axial movement of the OF. The used OF was without cladding.

### Light-assisted spinning procedure and optical setup

Glass syringes (10 ml) were used as the container for the PEO solution. The developed coaxial needle was integrated in an RT-Advanced (Linari engineering) electrospinning system. The coaxial needle was electrified by direct connection to the high voltage generator through metallic clip, while grounding the metallic collector. The chosen LED emitted at 520 nm and it was powered up through constant current driving circuit, giving 3 mW of optical power. LED optical power was measured with a custom made power meter. The light was guided to a lens system used to collimate the light at a controlled spot dimension. The light ray was then aligned and injected into the optical fiber through reflecting mirror mounted on a kinematic stand (see Fig. 3c). During testing, voltage ($V$) and solution flow rate ($\dot{Q}$) were independently set. The considered voltage range, in particular, was determined also based on solution concentration (see Figs. S3, S4, S5). Collector-needle distance was kept fixed at 20 cm. To extract the 2D-shape used for the image processing analysis and domain definition used in the numerical model the Taylor cone was observed and recorded digitally using CCD sensor through a long working distance zoom lens. A custom white LED matrix behind the drop provided the illumination to improve the image analysis and edge detection realized through customized MATLAB code (see also Video S1 in Supporting Information). For each ($V, \dot{Q}$) couple, pictures of the enlightened EF were taken through CCD reflex camera and nanofibers were deposited on an aluminum foil for a minimum of ten minutes before sample analysis (see Figs. S3, S4, S5). Pictures taken through CCD reflex camera were manually analyzed using ImageJ software to measure the illuminated length L. To ensure the repeatability of the test same light condition were maintained during the entire conducted test.

### Model-based feasibility studies

#### Numerical model

The numerical model was built through COMSOL Multiphysics software v5.5 (www.comsol.it). The droplet-jet transition region profile was modeled assuming axial symmetry as in Fig. 2a (upper sketch). In particular, point N denotes the base radius of the drop, F denotes the beginning of the EF, and O separates the drop region from the EF region. The distance of F from the axis was conventionally set to 10 µm, based on the characteristic initial diameter of the EF. The distance of N was set to 900 µm based on P1 and P2 data. The distance of O was conventionally set to 200 µm by considering the previous ones. The curved profile in between N and F was thus defined based on experimental recordings, while the EF rectangular domain was added for simulating light propagation up to a relatively far outlet (L_f_ = 20 mm, while drop-jet transition span is on the order of 1 mm). Refractive indices determining NA and light reflections/refractions were assumed fixed. The considered refractive index of the PMMA-OF is n_1_ = 1.4941 at the considered wavelength λ = 520 nm, while the surrounding air has a refractive index n_0_ = 1. The refractive index of the solution (n_2_ = 1.45) was established by literature^48^, since experimental parameters (high temperatures, variable concentration solute/solvent, etc.) affecting the measurement could not be controllable during the process. The maximum emission angle for the light rays exiting the OF (that constitute the emission cone) is ${\theta }_{NA}=arcsen \frac{\sqrt{{n}_{1}^{2}-{n}_{0}^{2}}}{{n}_{2}}$.

#### Analytical methods

The analytical method was developed on a customized Wolfram Mathematica’s code and based on the assumptions described in Taylor et al.^40^ considering the domains shown in Fig. 2c. The EF was considered with fixed radius r_EF_ = 10 µm (consistently with the numerical studies) and its axis was tilted at different angles (ϕ) respect to the OF axis spanning from 0° to 30°. In the first instance, longitudinal misalignments were not taken into account, but were reported as a case of study in the SI. A first frame x–y–z was placed at the corner of the emitting OF, while a second frame u–v–w was fixed at the center of the EF axis. The coordinate transformation was defined performing a roto-translation. Since the space between the OF and EF is assumed to be occupied by the droplet-jet transition zone (constituted by droplet and Taylor cone), the transformation between the two frames is mapped through the translation $\overrightarrow{t}$, whose z-component represents the width of this zone, while the y-axis represents the misalignment of OF’s axis respect to the EF’s axis. The u–v–w frame is also rotated through the $R$(ϕ) matrix describing the tilting of the jet during the drawing of the EF.

Only multimode and step index fibers, adopting the ray approximation in accordance with the experimental conditions, were adopted. The energy distribution was assumed uniform (in concordance with the numerical studies) because the OF was in overfilled launch condition, causing the energy density to be more concentrated near the fiber core axis. With the ray approximation assumptions, an analysis on the energy loss percentage coupled into the EF was simulated, concerning the ratio between the acceptable light area and the emitted one. To simulate this condition and following the approach of Taylor et al.^40^, a discrete set of circles with radius (${r}_{x}$) set from 0 to 250 µm and centered on the OF’s axis were considered (Fig. 2c). Any arbitrary point at the emitting-facet stands on the perimeter of a particular ring (${r}_{x}$), identifies a cone with an apex angle equals to $2{\theta }_{NA}$, whose origin is on the OF axis at $\frac{{r}_{x}}{tan({\theta }_{NA})}$ behind the x–y plane. The maximum acceptance angle for light rays entering the EF is ${\theta }_{EF}= arcsen \frac{\sqrt{{n}_{2}^{2}-{n}_{0}^{2}}}{{n}_{2}}$. The intersection between the emitting cones and the u-v plane containing the EF are ellipses (equations have been reported in the Supporting Information). Areas of these ellipses concern the energy potentially transmitted to the EF. In order to consider the percentage of the energy that is successfully transmitted, any ray (r) striking any point on the EF where is pointed an acceptance cone whose apex angle is equal or less than ${\theta }_{EF}$ was assumed consistent for the transmission. Equations and conditions to define loci of the limits of the acceptable light regions in the u-v plane have been reported in the SI.

### Nanofibers characterization

Nanofibers were collected on an aluminum foil wrap around the metallic grounded collector. After sample collection, nanofibers were gold sputtered (Quorum Q150R) and analyzed using FEI Helios NanoLab Dual Beam microscope (FEI Company: Hillsboro, OR, USA) . Collected SEM pictures of the obtained samples are reported in Supporting Information (Figs. S3, S4, S5). Nanofibers diameters were measured from image analysis using ImageJ software. Statistical analysis on each sample were performed using MATLAB, considering a minimum 30 measures for each sample. Data in Tables 3, 4were expressed as mean ± standard deviation. Subsequent correlation analysis was performed in MATLAB.

## Supplementary information

Supplementary Information.

Supplementary Video 1.

Supplementary Video 2.
