# Supplementary material for: Light-assisted electrospinning monitoring for soft polymeric nanofibers
Source: Sci Rep. 2020 Oct 1;10:16341. doi: 10.1038/s41598-020-73252-4 (PMC7529797; doi:10.1038/s41598-020-73252-4)
Supplement: Supplementary file 1 — Supplementary Information. [file 41598_2020_73252_MOESM1_ESM.pdf]

# Supporting Information

# Light-Assisted Electrospinning Monitoring for Soft Polymeric Nanofibers

Dario Lunni[1,2,3,*], Goffredo Giordano[1,2,3], Francesca Pignatelli[1], Carlo Filippeschi[1], Stefano Linari[4], Edoardo Sinibaldi[1,*] & Barbara Mazzolai[1,*]

[1]Center for Micro-BioRobotics
Istituto Italiano di Tecnologia
Viale Rinaldo Piaggio 34, Pontedera (PI) 56025, Italy

[2]The BioRobotics Institute
Scuola Superiore Sant'Anna
Viale Rinaldo Piaggio 34, Pontedera (PI) 56025, Italy

[3]Department of Excellence in Robotics & AI
Scuola Superiore Sant'Anna,
Piazza Martiri della Libertà 33, Pisa (PI) 56127, Italy

[4]Linari Engineering s.r.l.
Via Umberto Forti 24/14, Pisa (PI) 56121, Italy

E-mail:
dario.lunni@iit.it, dario.lunni@santannapisa.it
edoardo.sinibaldi@iit.it
barbara.mazzolai@iit.it

**NUMERICAL MODEL PROFILES**

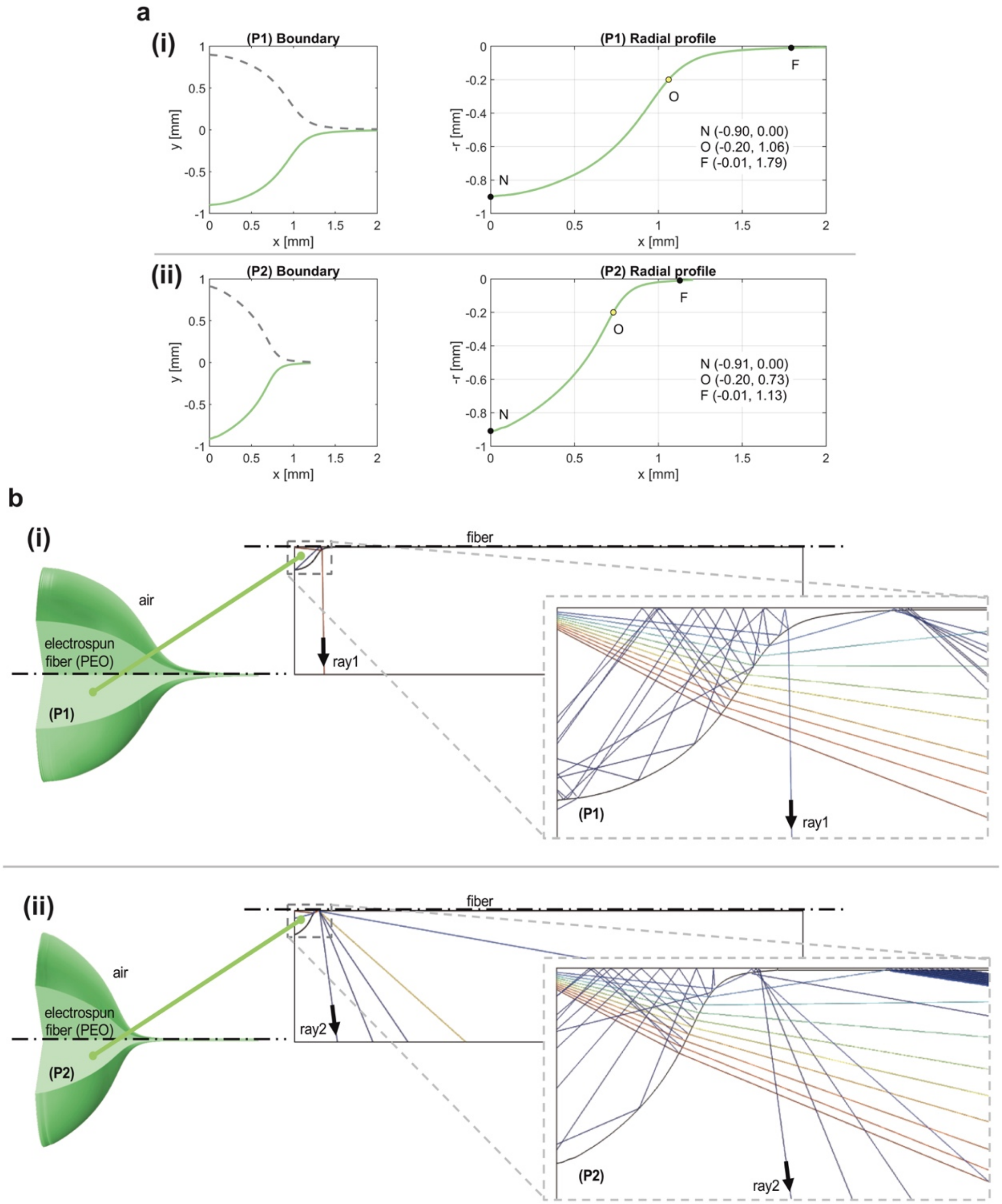


**Fig. S1** (a) Profiles derived through image processing from the experimentally recorded droplet-jet transition region. P1 profile correspond to a relatively good waveguide property of the electrospun fiber respect to P2 (L for profile P1 > L for profile P2). The boundary region is represented in (i). Assuming axial symmetry of the fiber only half of the profile was used to build the numerical model following the geometry in represented (ii). (b) Examples of resulting rays reflections and refractions obtained through the numerical models are represented for profile P1 (i) and P2 (ii).

## ANGULAR AND LONGITUDINAL MISALIGNMENT EVIDENCE

**Fig. S2** The angular misalignment of ∼25° is shown (worst case). Such deviation of the polymeric jet from a straight line comes as a consequence of the presence of a polymeric support used as scale to measure the distance through camera. The presence of such structure (as other possible disturbance) influences the symmetry of the electric field leading to the distortion of the electrospun jet.

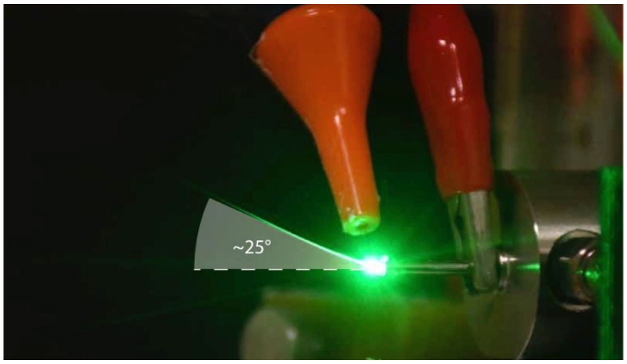

**EXPERIMENTAL SYSTEM VALIDATION: NANOFIBERS IMAGES AND LENGTH OF WAVEGUIDE LIGHT**

**Fig. S3-S4-S5** The tests used to validate the system usage are shown. The solutions at 8 %, 10 % and 12 % are tested varying voltage and solution flow until the electrospinning was taking place. The process results in terms of nanofiber SEM image and jet stream illumination are shown for each couple of parameters. In each picture a millimetric grid is shown at the bottom.

**Fig. S3**

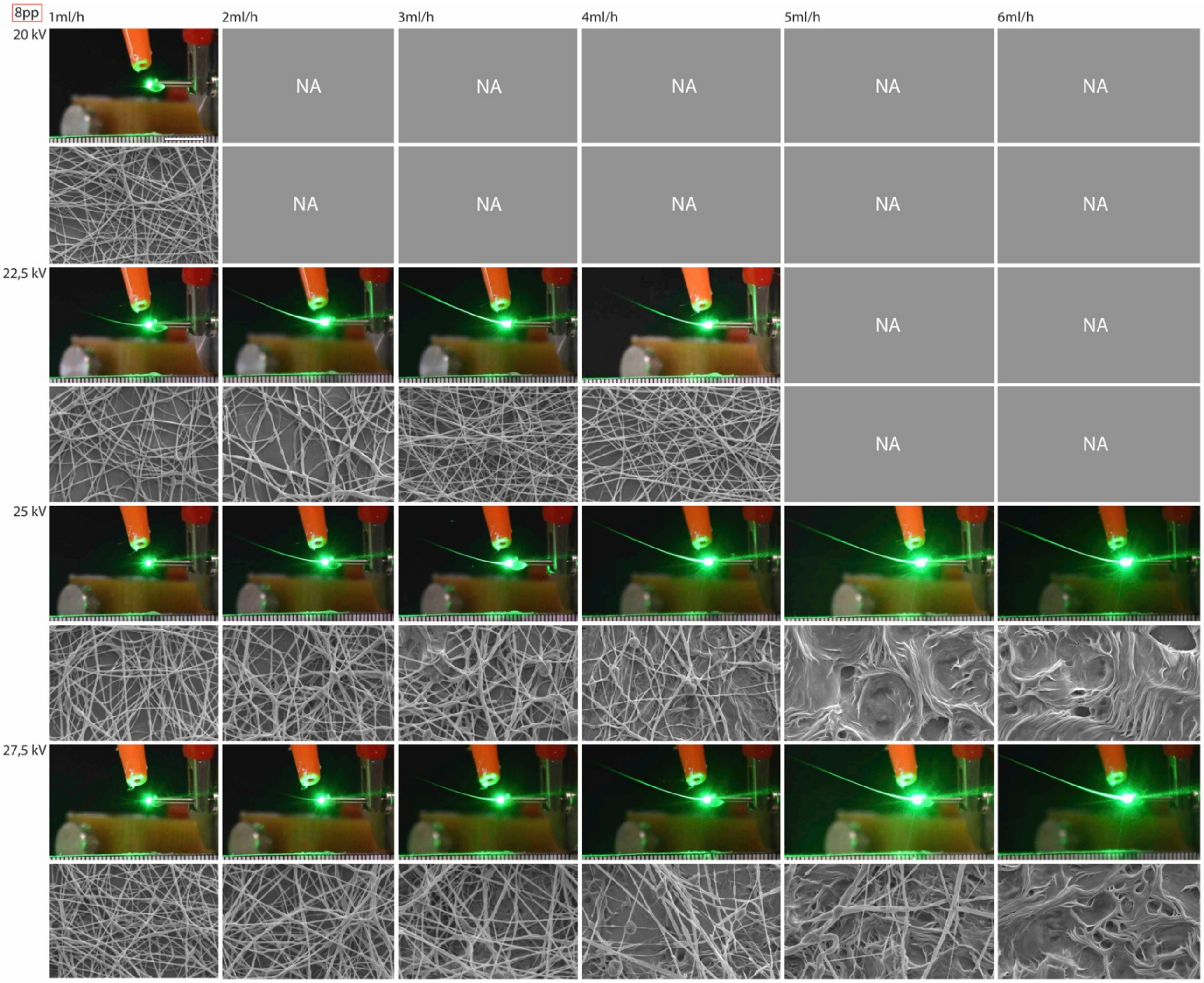

**Fig. S4**

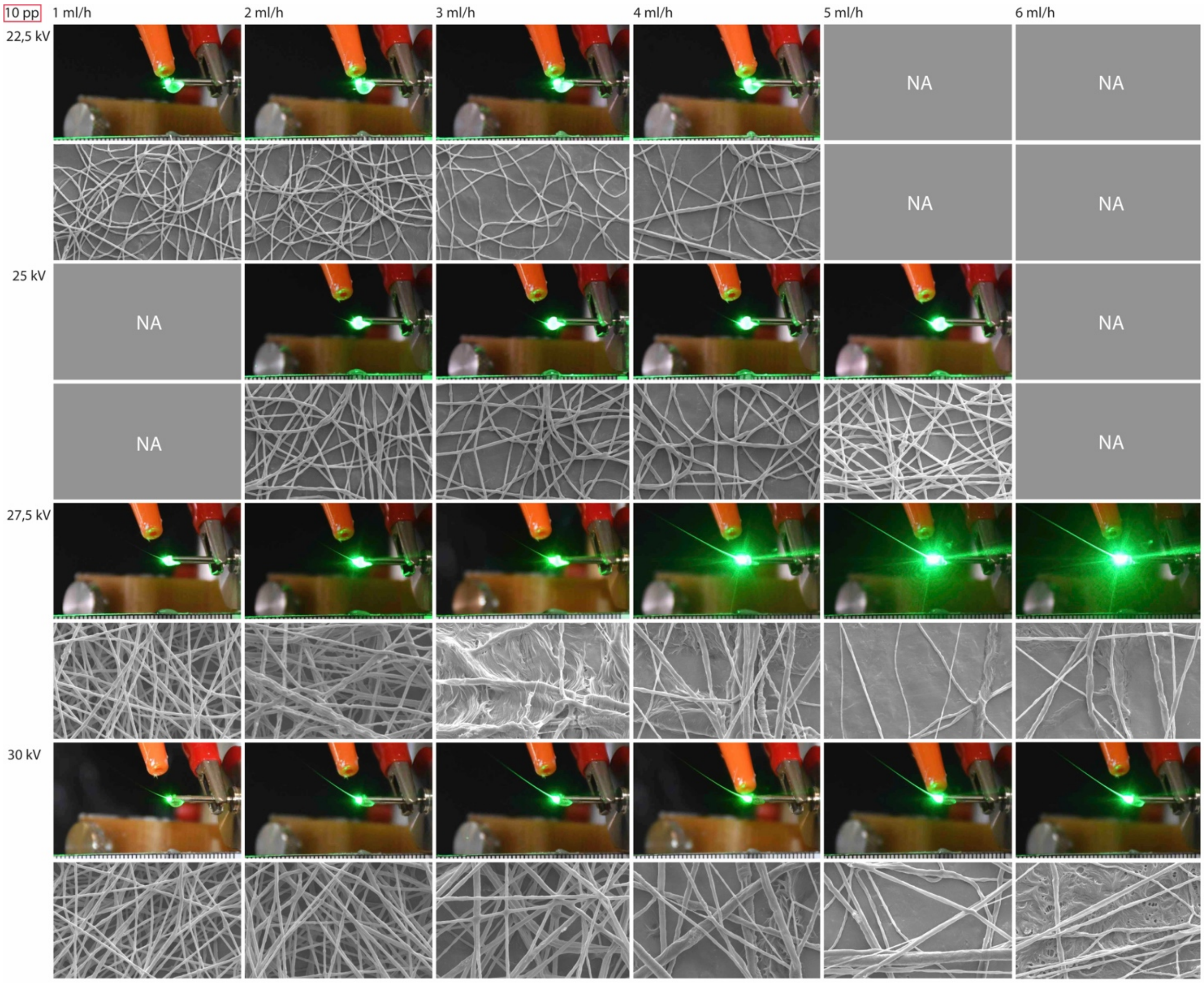

**Fig. S5**

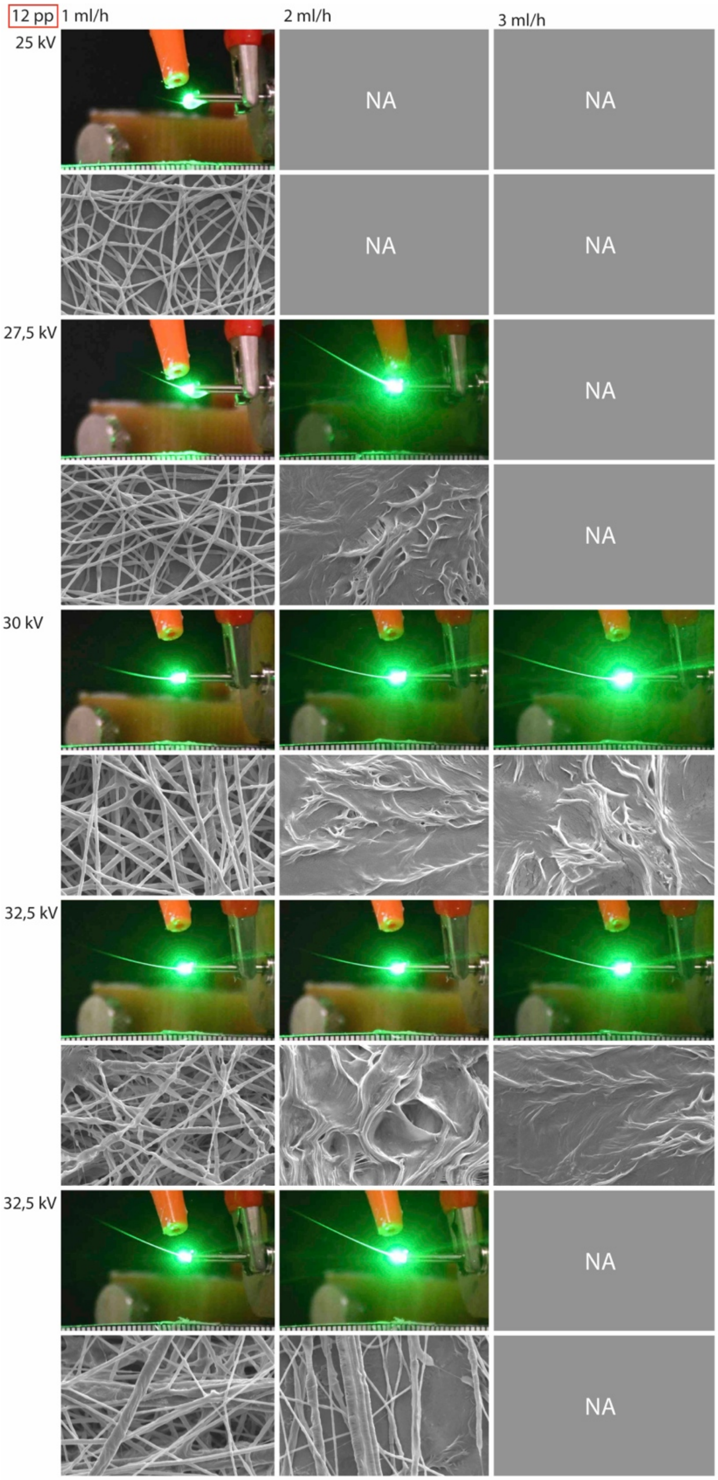

12 pp
1 ml/h
2 ml/h
3 ml/h
25 kV
27,5 kV
30 kV
32,5 kV
32,5 kV
NA
NA
NA
NA
NA
NA
NA
NA

**CORRELATION COEFFICIENTS**

Linear correlation coefficient among control process parameters ($C, \dot{Q}, V$) and output variables ($\widehat{D}, \sigma_D, \hat{L}$).

| | $C$ | $\dot{Q}$ | $V$ |
|---|---|---|---|
| $\widehat{D}$ | 0.5543 | 0.3242 | 0.6372 |
| $\sigma_D$ | 0.2048 | 0.3704 | 0.4500 |
| $\hat{L}$ | -0.2337 | 0.4220 | 0.3431 |

**LINEAR REGRESSION MODELS (Control parameters)**

Polynomial regression models combining control parameters variables ($C, \dot{Q}, V$) , until the second degree, were tested. The best fit in terms of $R^2_{adj}$ are here reported. The polynomial coefficient for each variable estimation along with the p-Value for each term are shown in the tables. Root mean square error, $R^2$ and $R^2_{adj}$ are also reported.

- $\widehat{D} = f_1(C, \dot{Q}, V)$

| | Estimation | p-Value |
|---|---|---|
| (Intercept) | -2365.8 | 0.008101 |
| $C$ | 400.19 | 0.025377 |
| $\dot{Q}$ | 28.821 | 0.01406 |
| $V$ | 22.388 | 0.0001661 |
| $C^2$ | -18.933 | 0.041305 |

Root Mean Squared Error: 79.3

R-squared: 0.699, Adjusted R-Squared: 0.659

- $\sigma_D = f_2(C, \dot{Q}, V)$

| | Estimation | p-Value |
|---|---|---|
| (Intercept) | -445.33 | 0.0065641 |
| $\dot{Q}$ | 36.824 | 0.0078941 |
| $V$ | 19.262 | 0.0020146 |

Root Mean Squared Error: 110

R-squared: 0.362, Adjusted R-Squared: 0.323

- $\hat{L} = f_3(C, \dot{Q}, V)$

| | Estimation | p-Value |
|---|---|---|
| (Intercept) | 18.073 | 0.0075294 |
| $C$ | -4.1393 | 0.0030107 |
| $\dot{Q}$ | 0.34249 | 0.00029428 |
| $V$ | 0.1131 | 0.0066288 |
| $C^2$ | 0.20128 | 0.0052518 |

Root Mean Squared Error: 0.581

R-squared: 0.598,  Adjusted R-Squared: 0.543

**LINEAR REGRESSION MODELS (Observable parameters)**

Linear regression model of $\widehat{D}$ and $\chi$ vs $\hat{L}$ (Fig. 4e-f ) are here reported along with $\mathrm{R}^2$.

$$\widehat{D} = g_1(\hat{L}) = a1 * \hat{L} + a2 \qquad \chi = g_2(\hat{L}) = b1 * \hat{L} + b2$$

| Concentration | a1 | a2 | $R^2[g_1]$ | b1 | b2 | $R^2[g_2]$ |
|---|---|---|---|---|---|---|
| 8 % | 44.64 | 180.3 | 0.53 | 0.077 | 0.29 | 0.34 |
| 10 % | 175.2 | 256.3 | 0.74 | 0.29 | 0.15 | 0.70 |
| 12% | 145.8 | 247 | 0.54 | 0.250 | 0.057 | 0.62 |

**ANALYTICAL METHOD EQUATIONS:**

- Theoretical background by Taylor et al.[1], and equations adopted.

Optical and geometrical experimental parameters :

$n_0$ = 1 ; (*refractive indexes*)
$n_1$ = 1.49 ;
$n_2$ = 1.45 ;

Numerical aperture of the Optical Fiber:

$\theta_{NA} = arcsen\ \frac{\sqrt{{n_1}^2 - {n_0}^2}}{n_2}$;
$k = \frac{1}{\tan(\theta_{NA})}$;

Optical Fiber radius:

$r_{OF} = \{0.1, 125, 250\}$ µm

$r_{claddingOF}$ ={0.1, 250}; µm (*considered from the fiber axis*)

Electrospun Fiber radius:

$r_{EF} = \{10\}$ µm

$r_{claddingEF}$ =10; µm (*considered from the fiber axis*)

Tilting angle:

$\phi = \{0, 5, 10, 15, 20, 25, 30\}°$;

Maximum accepting angle of the Electrospun Fiber:

$\boldsymbol{\theta_{EF} = arcsen\ \frac{\sqrt{{n_2}^2 - {n_0}^2}}{n_2}}$;
$k_2 = \frac{1}{\tan(\theta_{EF})}$;

$dz = \{500, 1000, 1500\}$; µm (*z-component of the translation $\vec{t}$*)
$dy = \{0,250,500\}$; µm (*y-component of the translation $\vec{t}$*)
$dx = \{0\}$; µm (*x-component of the translation $\vec{t}$*)

With reference to Fig. 2c the geometrical condition so that a conic section, of a generic emitting cone, on the u-v-w plane is an ellipse is:

$$\phi < 90° - \theta_{OF}°$$

1. Internal/external OF emission cone:

$g[u,v] = x^2 + (y - r_{claddingOF})^2 - (z - k\, \boldsymbol{r_{OF}})^2 / k$;
$i[u,v] = x^2 + (y - r_{claddingOF})^2 - (z + k\, \boldsymbol{r_{OF}})^2 / k$;

Coordinate change between the x-y-z and u-v-w reference system:

$x = u$;
$y = r_{claddingOF} \mathrm{Cos}[\Phi] - v \mathrm{Cos}[\Phi] + dy$;
$z = r_{claddingOF} \mathrm{Sin}[\Phi] - v \mathrm{Sin}[\Phi] + dz$;

Geometrical condition so that inner emission cone in the u-v-w plane is illuminated:

$$-\boldsymbol{k}\, \mathbf{r}_{\boldsymbol{OF}} + \mathbf{dz} - \mathbf{k_2 r}_{\boldsymbol{EF}} + \mathbf{r}_{\boldsymbol{claddingOF}} \mathbf{Sin}[\boldsymbol{\Phi}] - \mathbf{r}_{\boldsymbol{claddingEF}}\, \mathbf{Sin}[\boldsymbol{\Phi}] \geq \mathbf{0}$$

2. EF acceptable light receiving region traced by $u_0$'s, $v_0$'s roots:

$j[u_0, v_0] = (u - u_0)^2 + (v - v_0)^2 - w/k_2^2$;
$l[x, y] = x^2 + (y - r_{claddingOF})^2 - r_{OF}^2$;

Coordinate change:

$w = - \mathrm{Cos}[\Phi] (-dz - r_{claddingEF} \mathrm{Sin}[\Phi]) + \mathrm{Sin}[\Phi] (y - dy - r_{claddingEF} \mathrm{Cos}[\Phi])$;
$v = - \mathrm{Sin}[\Phi] (-dz - r_{claddingEF} \mathrm{Sin}[\Phi]) - \mathrm{Cos}[\Phi] (y - dy - r_{claddingEF} \mathrm{Cos}[\Phi])$;
$u = x$;

System of equations for constructing the acceptable light region using cones whose apex angle is equal to $\boldsymbol{\theta_{EF}}$, and are originated on the EF plane and are tangent to the emission ring in the OF plane.

1. $\mathbf{j}[\mathbf{u_0}, \boldsymbol{v_0}] = \mathbf{l}[\mathbf{x}, \mathbf{y}]$ (*First tangency condition: intersection between the emission ring $r_x = r_{OF}$ in the considered range of values and the ellipse (trace of the acceptance cone in the OF plane) after performing the above coordinate change*)

2. $\boldsymbol{\partial l[x, y]/\partial y = \partial\, j[u_0, v_0]/\partial y}$ (*Second tangency condition: considering the derivatives of the emission ring and the acceptance cone loci equal*)

Parametric equations to write down the locus of point ($u_0$, $v_0$) in the u-v plane:

$$\begin{cases} \mathbf{x} = \mathbf{r_{OF}}\, \mathbf{Sin}[\boldsymbol{\gamma}], & \boldsymbol{\gamma} \in [\mathbf{0°}, \mathbf{360°}] \\ \mathbf{y} = \mathbf{r_{claddingOF}} - \mathbf{r_{OF}}\, \mathbf{Cos}[\boldsymbol{\gamma}], & \boldsymbol{\gamma} \in [\mathbf{0°}, \mathbf{360°}] \end{cases}$$

- **Far-field:**

**Fig. S6**. Overlap of the emission (Blue) and acceptable (Cyan) light region in the EF (abscissa u, ordinate v) plane with the 10 um size EF core (Green), for a point source ($r_{c1} = 0, r_{cladding1} = 0$) originating at different distances dz = {500 um, 1000 um, 1500 um} on the OF-plane, at different longitudinal misalignment dy = {0

um, 250 um, 500 um} respect to the EF's axis and at fixed angular misalignment $\Phi = 0°, 10°, 25°$. Axes values reported are in um.

**Fig. S6.i**

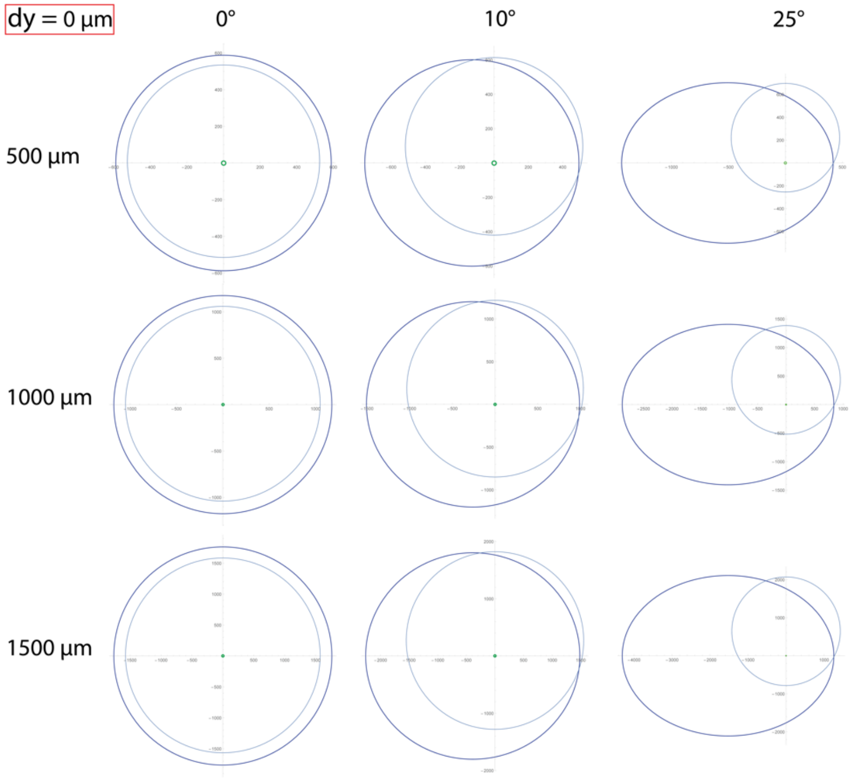

**Fig. S6.ii**

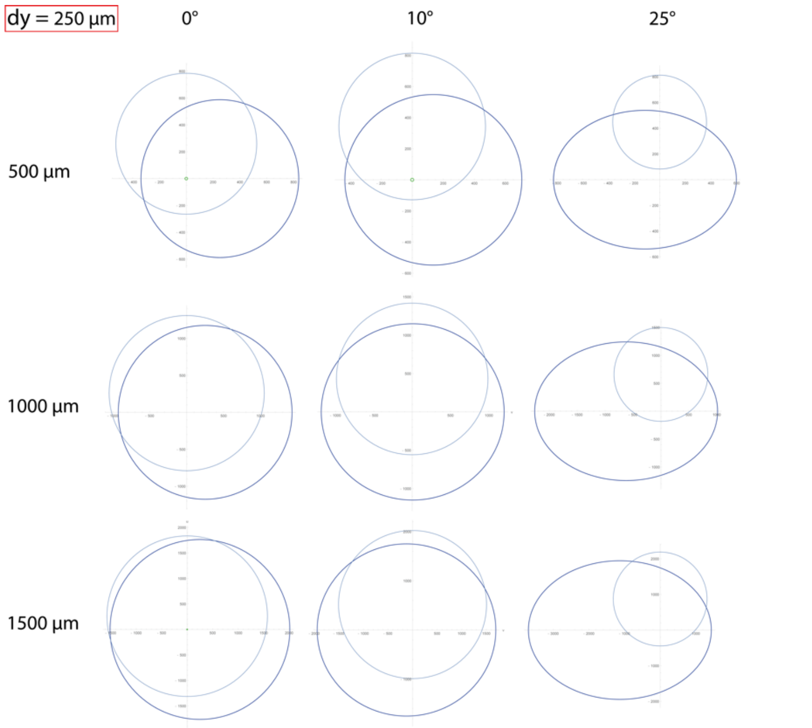


**Fig. S6.iii**

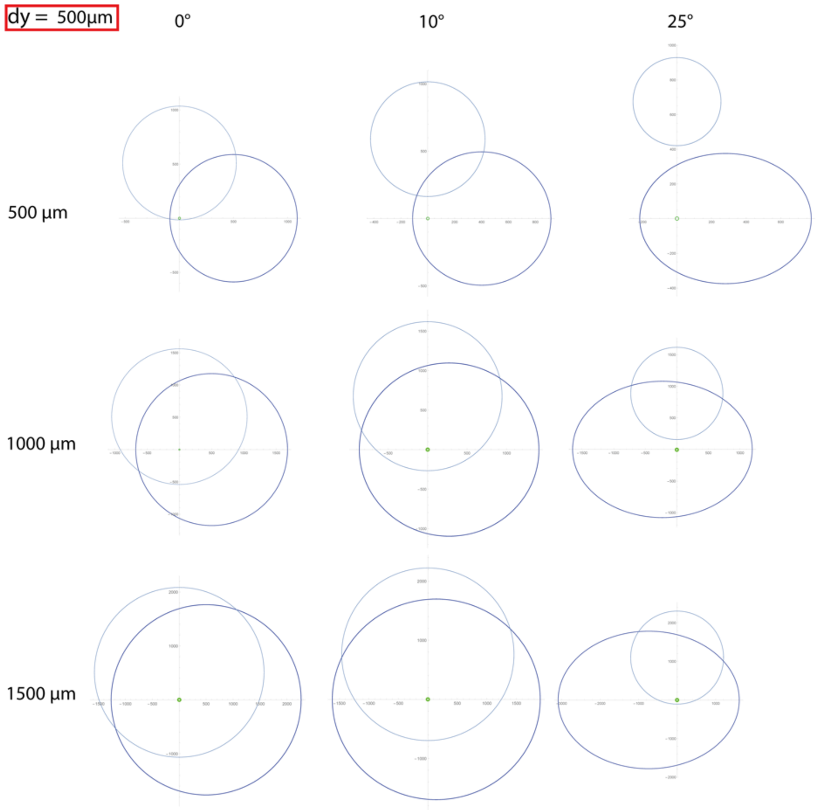

- **Near-field:**

**Fig. S7**. Accurate description has been accounted regarding the near-field case. To account a reasonable experimental condition, a silica-based ($n_{OF} = 1.4613$) step-index emitting fiber ($r_{OF}$ = 125 um, $r_{claddingOF}$ = 125 um) at small distances from the EF ($r_{EF}$ = 10 um, $r_{claddingEF}$ = 10 um, Green) has been modelled. The Taylor cone protrusion width is represented as 75 um, 175 um, 275 um thick, while the longitudinal misalignment is 35 um, 50 um and 100 um. The spanning angular misalignments are $\Phi = 0°, 10°$. In these conditions, the fiber's radii and the interlayer distances are on the same order. The emission fiber could not considered only as a point source. Emission (Blue) and acceptable (Cyan) light region in the EF plane are examined deeper than the far-field case. Uniform energy distribution is considered. As in the previous study, to define the limits of the acceptable light regions in the EF plane, are considered cones originating in the EF plane whose apex angle is equal to the critical $\theta_{EF}$, and tangent to the emission ring of the emitting fiber when $r_{OF}$ = $r_x$ = {0.1 um, 50 um, 125 um} is variable and the constraint on the domain is defined by the $r_{claddingOF}$ = 125 um. The emitted region has been considered as in the far-field case: in some cases the geometrical condition for the illumination of the inner emission cone is not satisfied and it is underlined by a cross symbol. The acceptable region discriminates how much energy has carried out into the EF. Therefore, to calculate the percentage of power accepted varying the angular and longitudinal misalignments, emission ring radii's solutions should be weighted. These tables report some cases where accepting regions coupled or not with the EF core. In general cross symbol depict not illuminated zones.

**Fig. S7.i**

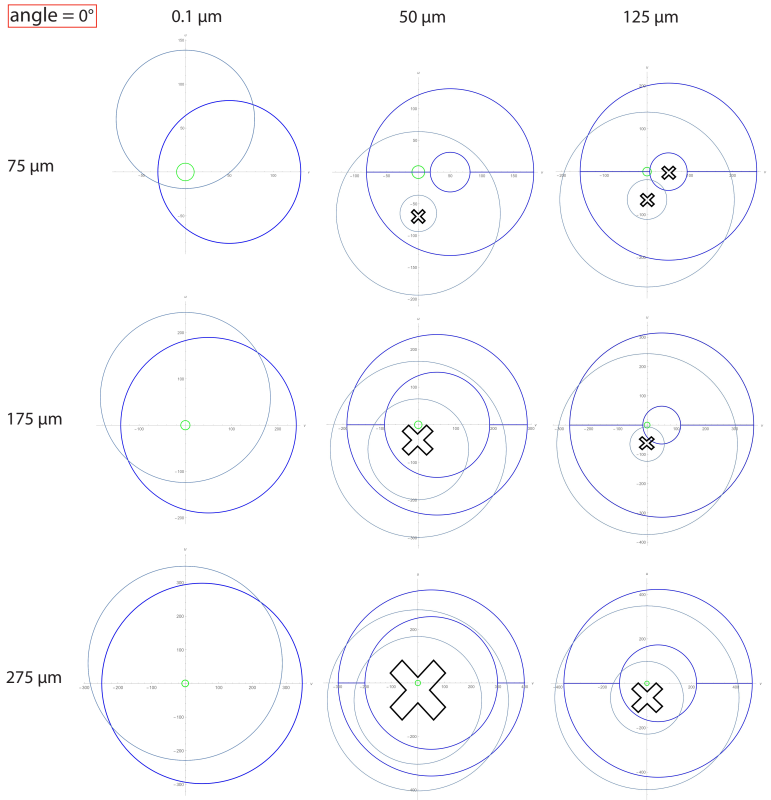

**Fig. S7.ii**

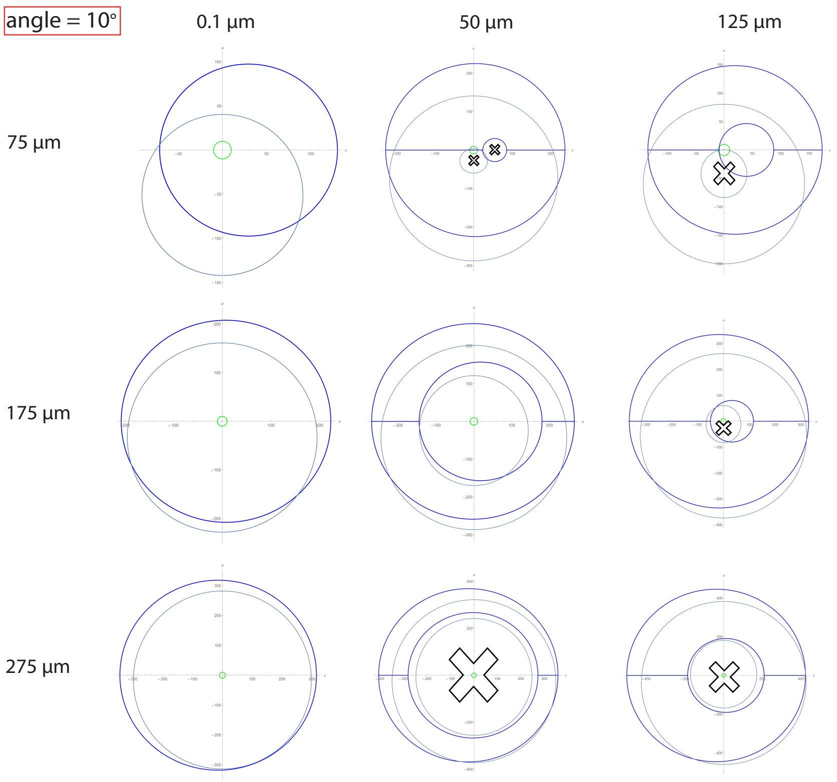


1. Taylor, K. M. & Anderson, B. L. Misalignment losses in fiber optic joints due to angular misalignment for arbitrary energy distribution. *Opt. Eng.* **34**, 3471–3479 (1995).

**VIDEO S1.** The edge detection system used to reconstruct the droplet shape is shown for two different working conditions (video not in real time). The contrast increase has been realized pointing a custom-made matrix of 4 low power LEDs toward the CCD sensor focused on the drop.

**VIDEO S2.** The light injection system in the electrospun fiber is shown for two different working conditions (video in real time). The different observable length of waveguide light is clearly visible when the droplet shape change.
